# No trade‐offs in interspecific interference ability and predation susceptibility in newt larvae

**DOI:** 10.1002/ece3.4465

**Published:** 2018-08-19

**Authors:** Monika Hloušková, Monika Balogová, Veronika Kršáková, Lumír Gvoždík

**Affiliations:** ^1^ Department of Botany and Zoology Masaryk University Brno Czech Republic; ^2^ Institute of Biology and Ecology Faculty of Science P.J. Šafárik University Košice Slovakia; ^3^ Institute of Vertebrate Biology of the Czech Academy of Sciences Brno Czech Republic

**Keywords:** amphibians, interspecific aggression, predator‐prey interaction, somatic growth, species coexistence

## Abstract

Coexistence of species with similar requirements is allowed, among others, through trade‐offs between competitive ability and other ecological traits. Although interspecific competition is based on two mechanisms, exploitation of resources and physical interference, trade‐off studies largely consider only species’ ability to exploit resources. Using a mesocosm experiment, we examined the trade‐off between interference competition ability and susceptibility to predation in larvae of two newt species, *Ichthyosaura alpestris* and *Lissotriton vulgaris*. In the presence of heterospecifics, *L. vulgaris* larvae slowed somatic growth and developmental rates, and experienced a higher frequency of injuries than in conspecific environments which suggests asymmetrical interspecific interference. During short‐term predation trials, *L. vulgaris* larvae suffered higher mortality than *I. alpestris*. Larvae of the smaller species, *L. vulgaris*, had both lower interference and antipredator performance than the larger *I. alpestris*, which suggests a lack of trade‐off between interference competition ability and predator susceptibility. We conclude that interference competition may produce a positive rather than negative relationship with predation susceptibility, which may contribute to the elimination of subordinate species from common habitats.

## 1 INTRODUCTION

Local coexistence among taxa sharing the same resources may occur through trade‐offs between their ecological traits. This proposition of ecological theory (Chase, 2011; Chesson, 2000; Levin, 1970) is based on the notion that species within a community are not superior in all ecological traits, such as competitive ability, predation vulnerability, stress tolerance, or colonization ability (Chase & Leibold, 2003; Kneitel & Chase, 2004). In fact, better performance of a given species in one trait is frequently counterbalanced by a lowered performance in other trait(s) and vice versa. Ecological trade‐offs have been widely reported in various aquatic and terrestrial communities (e.g., Bestelmeyer, 2000; Cadotte et al., 2006; Kraaijeveld & Godfray, 1997; Morin, 1983; Wellborn, 2002). Although trade‐offs between some ecological traits have received considerable attention, others are surprisingly understudied.

An example of a little‐known trade‐off is the relationship between interference competition ability and predation susceptibility. Interference competition, unlike exploitation of limited shared resources, is physical interference among interacting individuals using biting or producing harmful chemicals over access to a resource (Amarasekare, 2002; Grether, Losin, Anderson, & Okamoto, 2009). Its negative effect on interacting individuals is often highly asymmetric (Wellborn, 2002; Wissinger & McGrady, 1993; Wissinger et al., 1999). While the trade‐off between exploitative competition ability and predation susceptibility has been extensively studied on both a theoretical and empirical basis (Holt, Grover, & Tilman, 1994; Leibold, 1996; Skelly, 1995; Peacor & Werner, 2001; Kuang & Chesson, 2008; but see Murrell & Juliano, 2013), how interference competition is linked with predation susceptibility is little investigated (Cothran, Henderson, Schmidenberg, & Relyea, 2013). This is a nontrivial issue because interference competition is common among species (Grether et al., 2009). In addition, the ecological and evolutionary consequences vary substantially between both types of interspecific competition (Amarasekare, 2002; Delong & Vasseur, 2013; Grether et al., 2009; Holdridge, Cuellar‐Gempeler, & terHorst, 2016), which may also include the relationship with predation susceptibility.

Interference competitors may be affected by predators depending on actual interference mechanism, that is, territoriality, overgrowth, or allelopathy, which vary considerably among species (Amarasekare, 2002). For example, body size frequently determines the outcome of both interference competition and predation (Parker, 1974; Persson, 1985; Zhang, Andersen, Dieckmann, & Brannstrom, 2015). If larger species actively defend sites with the highest resources or safety from predators, they will be less exposed to predators than members of smaller species, which are expelled to less favorable sites. If the predator preferentially preys on smaller prey (Brodie & Formanowicz, 1983; Travis, Keen, & Juilianna, 1985), the susceptibility to predation further increases in the subordinate species. This scenario suggests no trade‐off between interference competition ability and predation vulnerability. On the other hand, exploitation and interference may be linked mechanistically by movement. Higher locomotor activity increases foraging rates, interaction with competitors, and perhaps with their predators (Delong & Vasseur, 2013). If both types of competition are linked, superior competitors should be more vulnerable to predators and vice versa. Clearly, more empirical results are needed to understand the relationship between interspecific interference competition and predation.

Salamander larvae and their predators are a suitable study system to examine the interference competition‐predation trade‐off. Unlike frog larvae, which often live in aggregations, salamander larvae are mostly solitary (Wells, 2007). They are aggressive toward other individuals, and so intraguild predation and cannibalism are common in many species (Anderson & Semlitsch, 2016; Griffiths, Dewijer, & May, 1994; Harris, 1987; Kishida et al., 2011). Salamander larvae also have many predators, making species’ coexistence in a given habitat depend not only on their interference ability but also on susceptibility to predation. However, whether they trade their interference ability for predation susceptibility is unknown.

**Figure 1:**
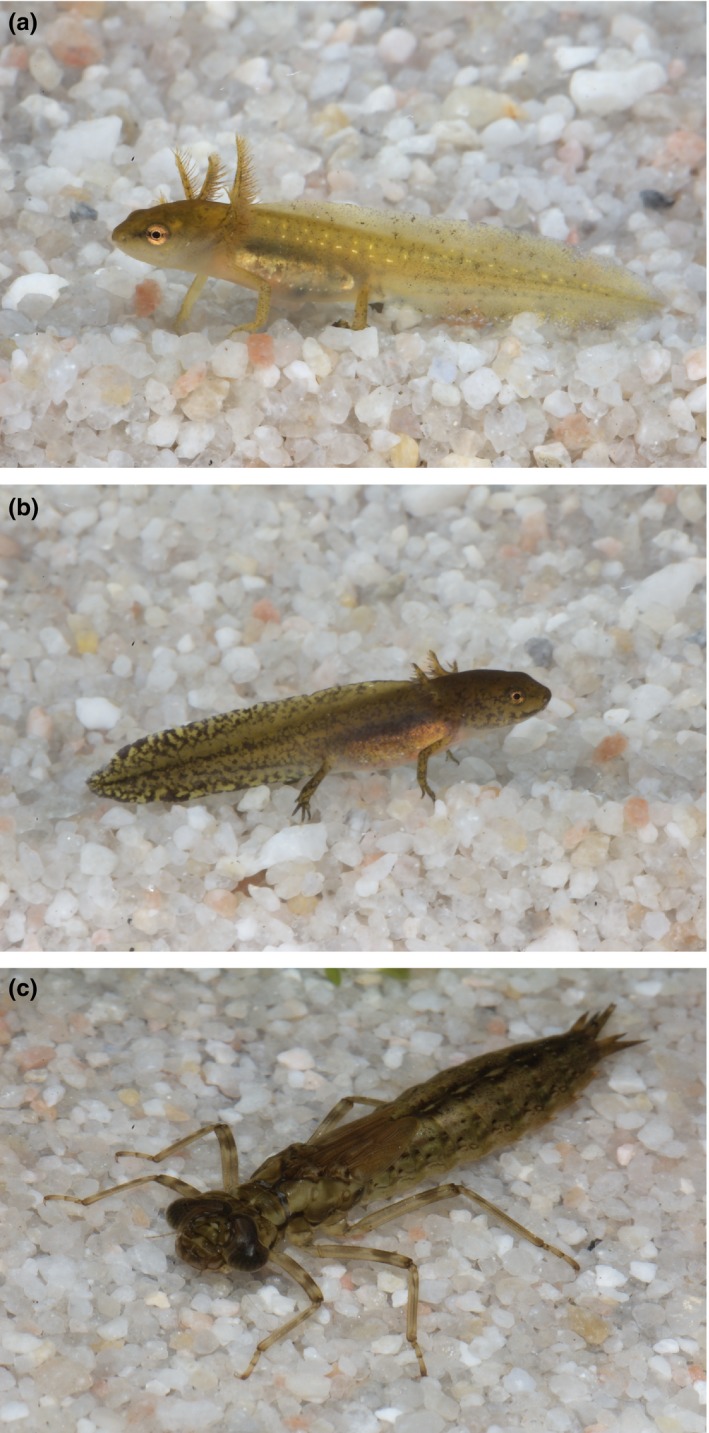
Study system. (a) Larva *Lissotriton vulgaris*, (b) larva *Ichthyosaura alpestris*, and (c) larva *Aeshna cyanea* [Colour figure can be viewed at http://wileyonlinelibrary.com]

We examined this trade‐off using larvae of two newt species, *Ichthyosaura alpestris* and *Lissotriton vulgaris*, and their common predator dragonfly *Aeshna cyanea* larvae, as a model system (Figure 1). Although these newt larvae vary in their size at hatching and metamorphosis (Van Buskirk, 2007), their body sizes largely overlap during larval development (Braz & Joly, 1994; Kuzmin, 1991; Szymura, 1974), which provides conditions for competition on their common food resource. Indeed, exploitation competition has been demonstrated between newt larvae of various species (Van Buskirk, 2007). In addition, injury rates vary among species within larval guilds (Szymura, 1974), even under the absence of predators (Vogrin, 2006). Because interspecific predation is absent between these taxa at the larval stage (Babik, 1998), naturally occurring injuries suggest, among others, some interference competition in newt larvae. Unfortunately, empirical evidence for interference competition is lacking.

We performed a mesocosm experiment, in which we subjected larvae of both species to competitive and predator‐prey interactions. The aim of this study was twofold. First, we asked whether injury rates vary, when larvae of both species were reared separately and together. Second, we examined whether the dominant species in interspecific interference competition would experience lower predation rates than subordinate species and vice versa. We predicted asymmetric interspecific competition in favor of *I. alpestris* over *L. vulgaris,* because the former is larger in body size (Persson, 1985). Also, if dragonfly larvae preferably prey on smaller newt larvae (Gvoždík & Smolinský, 2015), *L. vulgaris* larvae should be more susceptible to predation than the other species. Accordingly, this scenario predicts no trade‐off between competition and predation. However, larger *I. alpestris* larvae should be more active foragers to cover their higher energy demands, which increase their visibility to visually oriented predators (Werner & Anholt, 1993; Werner & McPeek, 1994). In this case, the competitive ability should be negatively associated with predation susceptibility in this system.

## 2 MATERIALS AND METHODS

### 2.1 Study system

Smooth, *L. vulgaris,* and Alpine, *I. alpestris,* newts are medium‐sized (total length 11 and 12 cm, respectively) tailed amphibians. They are common newt taxa widely distributed across most of Europe. Their geographic and altitudinal distributions largely overlap in Western, Central, and South‐Eastern Europe (Speybroeck, Beukema, Bok, Voort Van Der, & Velikov, 2016), and so they occur not only sympatrically but often syntopically in a variety of standing waters (see references above). The breeding period of both species highly overlaps (Szymura, 1974). During the spring (April‐June), females lay up to 300 eggs. Eggs are laid not in one clutch but individually onto leaves of aquatic vegetation (Díaz‐Paniagua, 1989). This unique oviposition behavior prolongs the individual oviposition period to several weeks. Newt larvae are strictly carnivorous. Animal plankton are a major part of their diet. Newt larvae of both have similar diet niches (Kuzmin, 1991). Larval development usually lasts until late summer. In cooler areas, larvae overwinter and finish metamorphosis during the next spring. In small water bodies, various dragonfly larvae, such as Southern hawker, *A. cyanea*, are the most important predators of newt larvae (Van Buskirk & Schmidt, 2000).

Newt larvae were obtained for experiments from eggs of wild‐caught females (*n *=* *15 for each species). Adults were captured from populations of *I. alpestris* (49°23′18″N, 15°30′48″E; 600 m) and *L. vulgaris* (49°22′56″N, 15°33′10″E; 560 m) near Jihlava, Czech Republic, located 4 km apart. Eggs were incubated in separate tanks under identical thermal (10.8 ± 4.1[*SD*]°C) and light (12.4 ± 19.9 klx) conditions, as in original tanks. Three to 5 days after hatching, free swimming larvae were transferred to two tanks (one per species), where they were mixed together. We assumed that the number of females sufficiently covers genetic variation in a given population. Larvae were haphazardly captured, photographed from the dorsal view using a digital microscope (magnitude 8×; DinoLite Pro, AnMo Electronics, New Taipei City, Taiwan), and distributed among experimental tanks. Overwintered dragonfly larvae (*n *=* *30) were captured from the same pools as adult newts.

### 2.2 Competition experiment

We used 30 fibreglass tanks (90 × 63 × 47 cm) for testing the influence of heterospecifics on larval traits. To reduce the effect of spatial variation on treatments, that is, absence or presence of heterospecifics, tanks were grouped into ten blocks (randomized block design) containing all treatment combinations. Tanks were located outdoors in a semi‐shaded area (water surface temperature = 17.5 ± 4.8[*SD*]°C; light intensity = 7.4 ± 15.9 klx). Each tank was initially filled with 90 L of nonchlorinated well water 2 weeks before releasing larvae. Following the previously published protocol (Van Buskirk & Schmidt, 2000), we added 12 g of dry beech soil and 3 g of fine hay to encourage algal and plankton growth. Each tank was inoculated with one liter of pond water and plankton. Several stems of aquatic plants (*Egeria densa*) and dry beech leaves were provided to act as hiding substrate for the larvae. Four snails (*Lymnea stagnalis*) were added to each tank to promote nutrient‐cycling. All tanks were covered with a fine mesh to prevent egg laying of insect predators.

Because the presence of predator cues induces plastic responses, increasing an individual's chance to avoid predation (Van Buskirk & Schmidt, 2000), each tank was equipped with a floating tube containing a dragonfly larva. Previous studies demonstrated that dragonfly larvae are important predators of newt larvae in our study population (Gvoždík & Smolinský, 2015; Smolinský & Gvoždík, 2013). To provide newt larvae with both predator odor and diet cues (Mitchell, Bairos‐Novak, & Ferrari, 2017), we fed dragonfly larvae with two living newt larvae at 4‐day intervals. The species composition of dragonfly prey was chosen according to species composition and density of newt larvae in a given tank, that is, only *I. alpestris*, only *L. vulgaris*, and mixed *I. alpestris*‐*L. vulgaris* diet. Dragonfly larvae were rotated randomly among tanks at weekly intervals.

Newt larvae (*n *=* *450 per species) were haphazardly distributed among tanks (*n *=* *10 per treatment) according to the absence or presence of heterospecifics, that is, only *I. alpestris* (30 individuals per tank), only *L. vulgaris* (30 individuals per tank), and *I. alpestris* together with *L. vulgaris* (15 *I. alpestris* and 15 *L. vulgaris*). Starting densities were chosen at the upper half of the natural densities range (Van Buskirk & Schmidt, 2000) to increase the frequency of individual interactions. We intentionally chose the replacement design, that is, keeping larval density at the same numbers in all tanks and changing the relative frequency of each species according to treatment, to maintain larval density, and thus the probability of individual encounters, at similar levels between treatments at the beginning of experiment (Smith, 1990). Although replacement design may bias the effect of intra‐ and interspecific competition, this bias is low in the case of the asymmetric interspecific competition (Underwood, 1997) we expected in this system. Because interspecific physical interference depends on the density (Semlitsch & Reichling, 1989) rather than on total biomass of each species, we composed heterospecific group according to number of larvae rather than their body mass.

After 30 days (20–28 June), all surviving larvae were recaptured using aquarium dipnets. We photographed (magnification 8×; DinoLite Pro, AnMo Electronics) each larva from the dorsal side and recorded developmental stage (Watson & Russell, 2000) and the presence of injuries, such as missing legs or damaged tail fins. From digital photographs, we measured larval total length (TL) from the tip of snout to end of tail (resolution 0.001 mm) using DinoCapture 2.0 (AnMo Electronics) software. Using these data, we calculated mean somatic growth rate as (mean final TL – mean initial TL)/duration of experiment (30 days), for each tank and species. Survival rate was calculated as the number of survivors/number of individuals at the start of the experiment, in each tank and species.

### 2.3 Predation trials

We performed predation trials following the previously published experimental protocol (Smolinský & Gvoždík, 2013). We used ten plastic aquaria (50 × 30 × 18 cm) filled with 18 L of tap water. To avoid the confounding influence of habitat complexity (Kopp, Wachlevski, & Eterovick, 2006), each aquarium contained only a datalogger (see above) that recorded water temperatures and light intensity at hourly intervals. Aquaria were covered with a meshed lid. Randomly oriented aquaria were placed in an outdoor area. Predation trials were performed at 16.8 ± 3.7[*SD*]°C and 13.1 ± 15.2 klx 2 days after finishing the competition experiment in a given block.

We placed haphazardly chosen newt larvae (*n *=* *10; TL: *I. alpestris* = 31.7 ± 2.4[*SD*] mm; *L. vulgaris* = 29.3 ± 2.0 mm) at the same developmental stage (the fifth toe clearly visible) into aquaria for 12 hr before beginning experiments (experiment start time 8:00). The trial group contained larvae of one species from the conspecific group only to avoid the confounding effect of interspecific interference on predation susceptibility. All chosen larvae were uninjured, because tail damage affects predation susceptibility in amphibian larvae (Semlitsch, 1990). Larval density was chosen to maximize the number of predator–prey interactions during a trial. We then added one randomly chosen dragonfly nymph (TL = 36.2 ± 4.6[*SD*] mm) from the rearing tanks into the aquaria and left it undisturbed for 24 hr. We utilized short timeframes for the predation trial to eliminate confounding factors on prey escape velocity, such as developmental and plastic responses, and to prevent eradication of the whole group by a predator. Dragonfly larvae were starved for 3 days before a trial to control for differing hunger levels. Each dragonfly larva was used in one trial only. After each trial, the number of injured, killed, and eaten newt larvae was counted. Dragonfly larva always consumed all killed individuals. Between trials, water was changed in all tanks to remove olfactory cues from previous predation episodes.

### 2.4 Data analysis

We applied a semiparametric randomization approach (9999 permutations) for data analysis (Quinn & Keough, 2002), because the sample size used prevented determination of the distribution of measured variables. We modeled the influence of heterospecific interactions on growth, developmental, injury, and survival rate for each species separately to avoid artificially inflated degrees of freedom in heterospecific pairs. Besides the treatment factor, that is, the presence or absence of heterospecifics, each model contained the random factor, block, and mean TL of larvae from each tank as covariates. We included body size because it is an important determinant of interference competition (Zhang et al., 2015). Except growth rate, we used the final TL for this purpose. We applied a model reduction approach and removed factors that contributed little (variance components < 0) to the total explanatory value. To analyze the susceptibility of predation, that is, number of killed or injured larvae, the model included species identity as a fixed factor and block as a random factor. Predator length, mean prey length per trial, mean water temperature, temperature range, and mean light intensity were added as the model covariates. Note that reported *F*‐ratios are in fact pseudo‐values because permutation tests have no known distribution under a true null hypothesis. The frequency of injuries between treatments was tested using Fisher's exact test. Trait associations were examined using Spearman correlation coefficients. Corresponding *p*‐values were obtained using permutation tests (9999 permutations). Results are presented as means with 95% confidence intervals. Confidence intervals were calculated using the nonparametric bootstrap procedure (“bsa” method, 9999 replications). All analyses were performed in the PERMANOVA module of the Primer 6 package. Confidence intervals and correlation tests were calculated using the “boot” (Canty & Ripley, 2017) and “coin” (Hothorn, Hornik, van de Wiel, & Zeileis, 2006) packages, respectively, in R.

## 3 RESULTS

**Figure 2:**
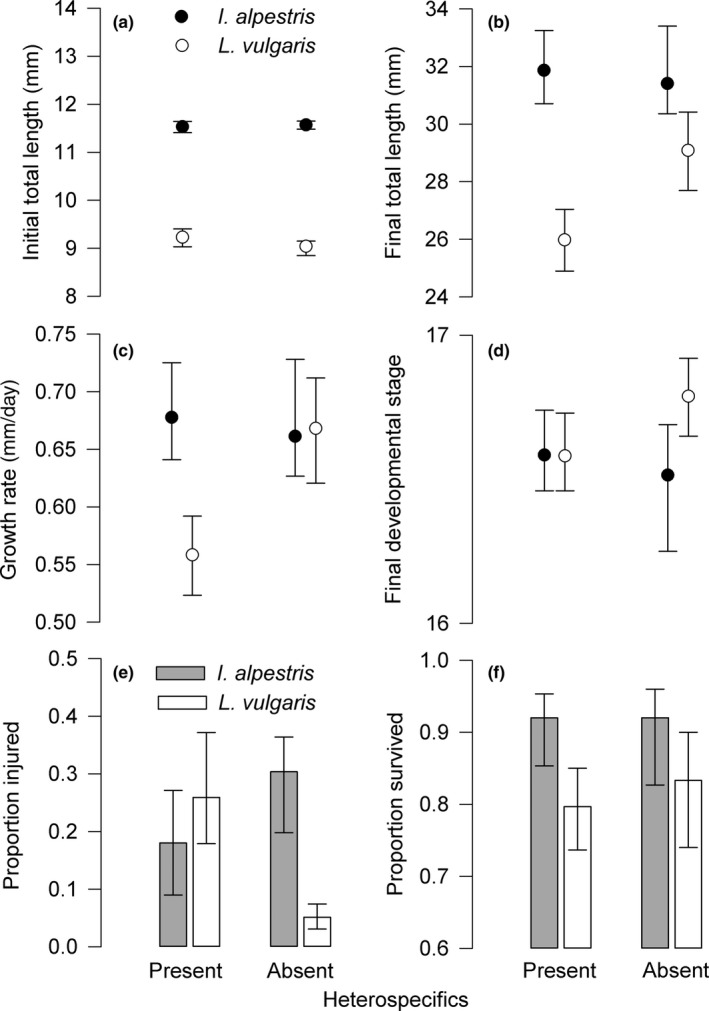
The influence of heterospecific interactions on (a) initial total length (mean per tank), (b) final total length, (c) somatic growth rate, (d) final developmental stage (after Watson & Russell, 2000), (e) proportion of injured individuals (damaged tail fins or missing limbs), and (f) proportion survived in larvae of two newt species, *Ichthyosaura alpestris* and *Lissotriton vulgaris*. All values are means ±95% CI. Growth rate means are corrected for the effect of initial total length. (*n *=* *15 per treatment and species). Legends in (a) and (e) pertain to all other graphs

At the beginning of the competition experiment, hatched *I. alpestris* larvae were on average 26% longer than *L. vulgaris* (*F*_1,9_ = 802.61, *p *<* *0.001; Figure 2a). Within species, larval TL was similar in both conspecific and heterospecific groups (*I. alpestris*:* F*_1,9_ = 0.41, *p *=* *0.55; *L. vulgaris*:* F*_1,9_ = 3.69, *p *=* *0.09). After 1 month, TL remained similar in both treatments in *I. alpestris* (*F*_1,9_ = 0.33, *p *=* *0.57; Figure 2b). In *L. vulgaris*, larvae from heterospecific tanks were 12% shorter than larvae from conspecific groups (*F*_1,9_ = 9.46, *p *=* *0.01; Figure 2b). Accordingly, *L. vulgaris* larvae in heterospecific groups grew slower than larvae reared under the absence of interspecific interactions (*F*_1,9_ = 9.46, *p *=* *0.01; Figure 2c). The effect of heterospecific interactions on larval developmental rate was statistically nonsignificant in *I. alpestris* (*F*_1,9_ = 0.49, *p *=* *0.50). In *L. vulgaris,* conspecific larvae developed faster than larvae in heterospecific groups (*F*_1,9_ = 9.73, *p *=* *0.02; Figure 2d).

**Figure 3:**
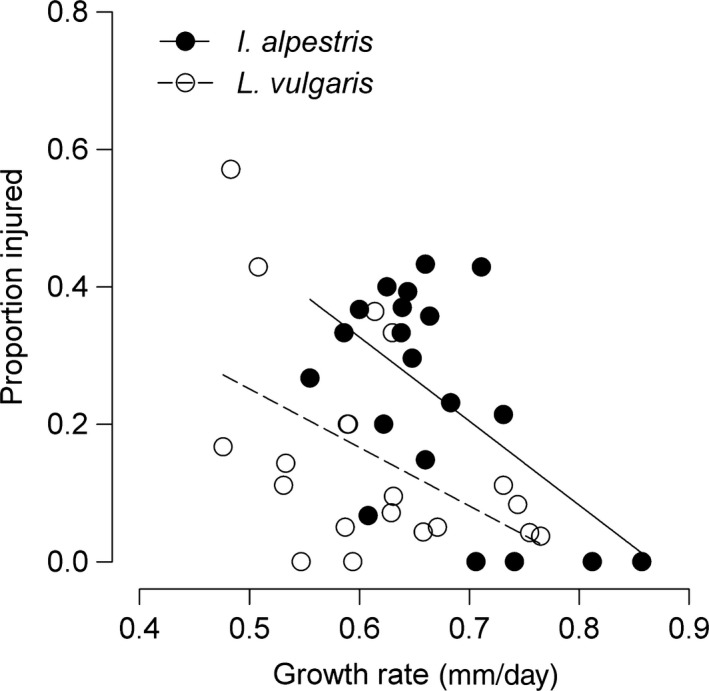
Association between somatic growth rate and the proportion of injured individuals in larvae of two competing newt species. Sample size (*n *=* *20 per species) includes values from both con‐ and heterospecific groups. Note that regression lines were added to illustrate trends only. See text for statistical results

The influence of heterospecifics on the proportion of injured larvae varied between species. In *I. alpestris*, interspecific competition had no detectable effect on the proportion of injured larvae (*F*_1,9_ = 2.64, *p *=* *0.14). Under the absence of heterospecifics, *L. vulgaris* larvae experienced a lower incidence of injuries than in their presence (*F*_1,9_ = 18.93, *p *=* *0.002; Figure 2e). The injuries were concentrated exclusively on tail fins and limbs. The frequency of injuries on tails and limbs was similar in both con‐ and heterospecific groups (*I. alpestris*: Fisher's exact test, *p *=* *0.73_;_ *L. vulgaris*:* p *=* *1). Overall, *L. vulgaris* experienced a higher proportion of missing limbs (22 of 66 injured larvae) than *I. alpestris* larvae (12 of 123; *p *<* *0.001). The frequency of injuries was negatively associated with growth rates in *L. vulgaris* (*r*_s_ = −0.49, *n *=* *20, *p *=* *0.03; Figure 3). The same trend in trait association was statistically nonsignificant in *I. alpestris* (*r*_s_ = −0.38, *n *=* *20, *p *=* *0.10). Despite the variation in injury frequencies, larvae in all groups survived at similar rates during the 30‐day trial period (*I. alpestris*:* F*_1,9_ < 0.001, *p *≈* *1; *L. vulgaris*:* F*_1,9_ = 0.49, *p *=* *0.53; Figure 2f). Spatial blocking had a statistically nonsignificant effect in all cases.

**Figure 4:**
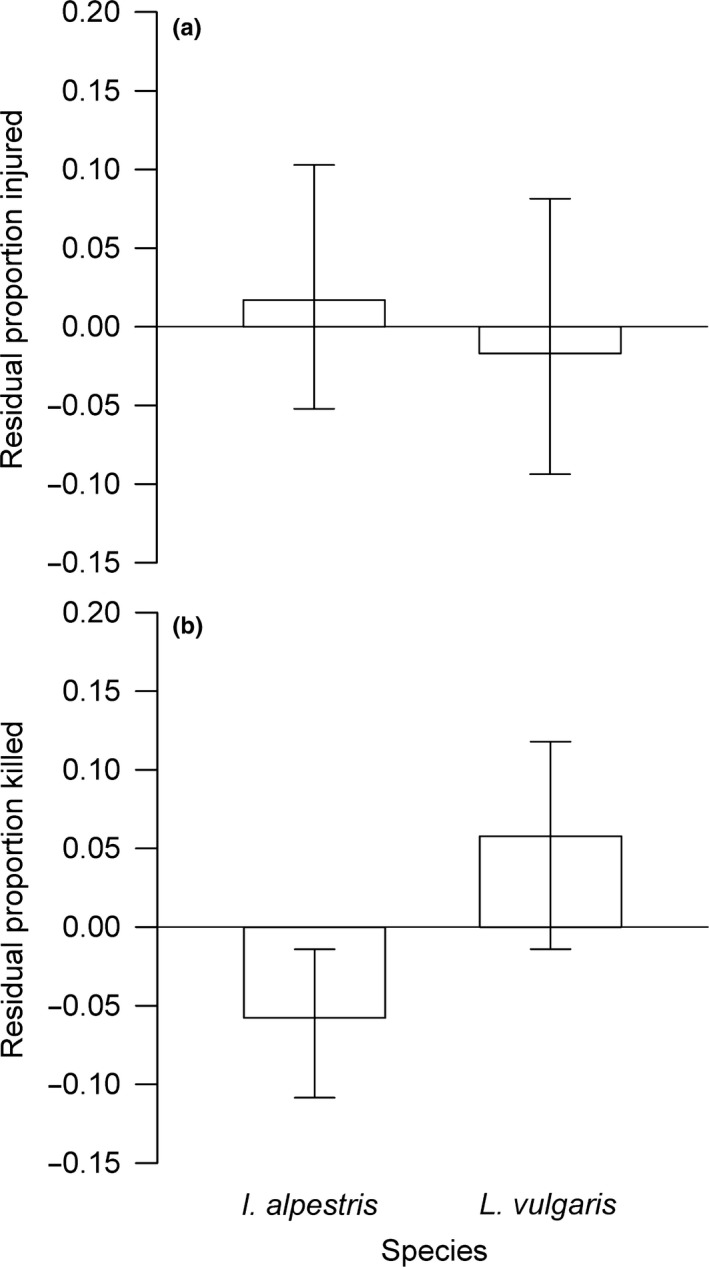
Proportion of (a) injured and (b) killed individuals after 24 h exposure to a predator (dragonfly larva) in two newt species. Values (mean ± 95% CI) are residuals from the relationship with predator total length. (*n *=* *20 per species)

**Figure 5:**
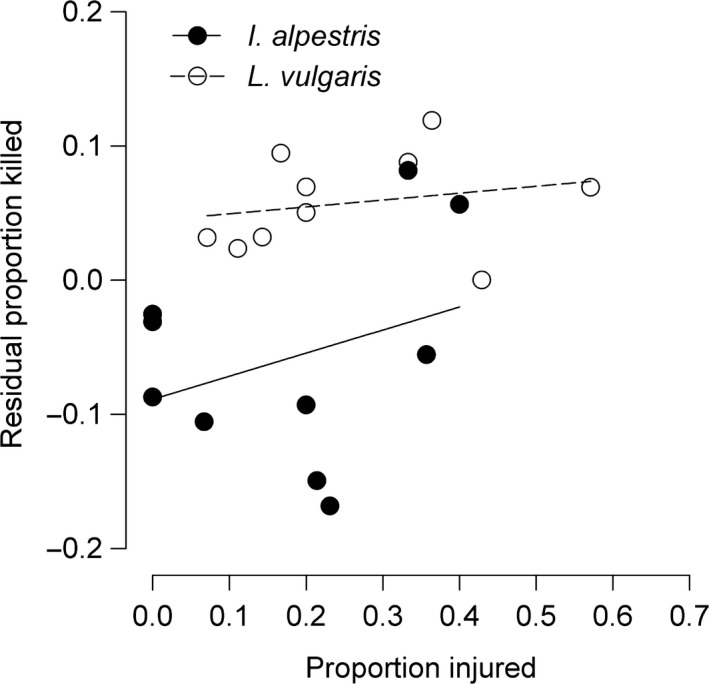
Association between the proportion of killed individuals from predation trials and the proportion of injured individuals from competition experiments in larvae of two competing newt species. Sample size (*n *=* *10 per species) shows values per block. Note that regression lines were added to illustrate trends only. See text for statistical results

We performed 40 predation trials (*n *=* *20 per species) using larvae from both species. The number of injured or predator‐killed larvae (up to 7 of 10 individuals) depended not on mean TL of newt larvae but on TL of dragonfly larva (Injuries: *F*_1,28_ = 4.30, *p *=* *0.046; Kills: *F*_1,28_ = 4.83, *p *=* *0.04). After correcting for predator body size, newt larvae of both species showed a similar frequency of injuries after a trial (*F*_1,28_ = 0.03, *p *=* *0.86; Figure 4a). The predator size‐corrected proportion of killed larvae was higher in *L. vulgaris* than in *I. alpestris* (*F*_1,28_ = 6.68, *p *=* *0.01; Figure 4b). We found no association between the proportion of killed larvae from predation trials and the proportion of injured larvae from the competition experiment in both species (*I. alpestris*:* r*_s_ = 0.15, *n *=* *10, *p *=* *0.68; *L. vulgaris*:* r*_s_ = 0.29, *n *=* *10, *p *=* *0.42; Figure 5).

## 4 DISCUSSION

In this study, we aimed (a) to provide evidence for interference competition as measured by injury rates and (b) to test for a trade‐off between interference competition ability and predation susceptibility between larvae of two newt species. The presence of heterospecifics reduced somatic growth and increased frequency of injuries in the smaller species, *L. vulgaris*. The reduced growth rate suggests an asymmetric effect of interspecific exploitation, whereas increased injury rates likely resulted from physical interference. Contrary to the competition‐predation trade‐off hypothesis, we found no association between the proportion of killed individuals from predation trials and the proportion of injured individuals from the competition experiment in both species. Overall, the subordinate species was more susceptible to predation by dragonfly larvae than the dominant species. This suggests no trade‐off between interference competition ability and susceptibility to predation in this system.

The presence of heterospecifics reduced growth rates in *L. vulgaris* larvae. The slowed growth of *L. vulgaris* larvae has also been reported in the presence of other newt species, *Triturus cristatus* (Griffiths et al., 1994). Generally, growth rate is an important fitness component of amphibian larvae (Werner, 1986). Faster growth allows larvae to escape gape‐limited predators (Urban, 2007), to provide advantage in physical interference (Smith, 1990), and to reach faster a minimum size at metamorphosis (Semlitsch & Caldwell, 1982). Given the importance of growth rates on larval fitness (but see Earl & Whiteman, 2015), this result suggests an asymmetric form of interspecific competition between these species.

The reduced growth rate in the subordinate species may result from two mutually nonexclusive causes. First, smaller *L. vulgaris* larvae were less successful in exploitation of resources than the larger *I. alpestris*, because consumption rate is in general body size dependent (Pawar, Dell, & Savage, 2012; but see Persson, 1985). Second, the reduced growth rate may result from physical interference, because subordinates were expelled from resource‐rich sites, and (or) injured larvae allocated energy to injury regeneration at the expense of growth (Lynn, Borkovic, & Russell, 2013; but see Starostová, Gvoždík, & Kratochvíl, 2017). The negative association between the frequency of injuries and growth rates may favor the second explanation. However, without additional information, we cannot discriminate the potential causes of slowed somatic growth in subordinate species.

In the presence of heterospecifics, *L. vulgaris* larvae experienced a higher frequency of injuries than in conspecific groups. In other systems injured appendages (i.e., tails, toes, or gills) among individuals have indicated interference competition within a population (Van Buskirk & Smith, 1991; Wellborn, 2002; Vervust, Van Dongen, Grbac, & Van Damme, 2009). In our study, the increased injury frequency in a subordinate species likely resulted from the aggressive behavior of the larger species, *I. alpestris,* which is the common mechanism of interspecific interference competition (Grether et al., 2013; Peiman & Robinson, 2010). Our anecdotal observations confirmed that *I. alpestris* larvae indeed attack other individuals if they approached too close, which is similar to aggressive behavior in other salamander taxa (Walls & Jaeger, 1987). Theory predicts that aggression should be stronger among individuals with a high resource overlap, that is, conspecifics, than among heterospecific counterparts (Peiman & Robinson, 2010). Previous studies showed that diet composition varies among species because of body size variation in newt larvae, which suggests a higher resource overlap within, rather than between species (Braz & Joly, 1994; Kuzmin, 1991; Szymura, 1974). Accordingly, intraspecific aggression should be stronger than interspecific interference. If injury rates correctly estimate aggression levels in newt larvae, our results provide no support for this prediction.

Alternatively, injuries may be interpreted as attempts of intraguild predation, because cannibalism and predation are common among competing salamander larvae (Anderson & Semlitsch, 2016; Griffiths et al., 1994; Harris, 1987; Kishida et al., 2011). Although stomach content analyses of newt larvae were unavailable, this explanation seems unlikely for several reasons. First, laboratory experiments showed no evidence for predation between the larvae of both species despite large differences in body size (Babik, 1998). Second, interactions between both species provided no apparent benefit, in terms of faster growth or higher survival, to the presumed intraguild predator (Polis, Myers, & Holt, 1989). Third, in contrast to other systems (Cothran et al., 2013), larval survival was similar in both con‐ and heterospecific groups despite variation in the frequency of injuries (Figure 1f). Hence, the ecological consequence of presumed nonlethal intraguild predation, that is, consumption of body parts without killing the competing prey, is indistinguishable from interspecific interference competition. Finally, larvae of both species experienced similar injury rates in heterospecific groups and the conspecifics of *I. alpestris*, which suggests that the higher injury rates in *L. vulgaris* likely resulted as a byproduct of intraspecific aggression in the dominant species (Peiman & Robinson, 2010).

During predation trials, *L. vulgaris* larvae were killed more frequently than *I. alpestris* larvae. The predation susceptibility of amphibian larvae to dragonfly larva generally depends on two prey attributes, body size and the magnitude of predator‐induced plasticity of behavioral and morphological traits (Arendt, 2009; Calsbeek & Kuchta, 2011; Gvoždík & Smolinský, 2015; Johnson, Burt, & DeWitt, 2008; Van Buskirk & Schmidt, 2000). In our study, *L. vulgaris* larvae were smaller than *I. alpestris*. The higher susceptibility to predation in the smaller species may result from size‐selective predation, because dragonfly larvae preferentially prey on smaller newt larvae (Gvoždík & Smolinský, 2015), or simply because their digestive system capacity allows consuming a higher number of smaller individuals than larger ones. Previously published results showed that the magnitude of predator‐induced plastic responses in morphological traits was lower in *L. vulgaris* than in *I. alpestris* (Schmidt & Van Buskirk, 2005). This suggests that the higher predation susceptibility in *L. vulgaris* relative to *I. alpestris* may result from both smaller body size and the limited magnitude of a predator‐induced plastic response.

Theory predicts that the absence of a competition‐predation trade‐off should accelerate the elimination of the subordinate species (Chase & Leibold, 2003). Hence, the frequent coexistence of both newt dragonfly taxa in natural habitats (Kuzmin, 1991; Szymura, 1974; Van Buskirk, 2007; Van Buskirk & Schmidt, 2000; Vogrin, 2006) should be enabled by other factors than competition–predation trade‐off during larval development. Breeding periods highly overlap between both species (Speybroeck et al., 2016), and so its contribution to reduced competition between their larvae seems negligible. Limited interspecific variation in consumed food and microhabitat use (Kuzmin, 1991; Szymura, 1974) also suggests that these niche dimensions cannot explain species coexistence in this system. Other authors propose that both species co‐occur because the prolonged oviposition period (see above) produces different‐sized larvae, which accordingly decreases their intra‐ and interspecific competition (Szymura, 1974; Fasola 1993; Vogrin, 2006). However, body size differences contribute little to resource partitioning in newt larvae under seminatural conditions (Van Buskirk, 2007). Hence, factors affecting the co‐occurrence of both species remain to be determined.

From our studies, we propose an additional two candidate trade‐offs enabling the coexistence of both species. The first trade‐off is between competitive ability and thermal stress tolerance. Adult *L. vulgaris* prefer higher body temperatures than *I. alpestris* (Balogová & Gvoždík, 2015). Our unpublished data confirm the same pattern in larvae (B. Winterová and L. Gvoždík, unpublished data). So, the competitively inferior *L. vulgaris* may have a competitive advantage at higher water temperatures than those in our study. This is also consistent with the habitat use of both species. *Lissotriton vulgaris* commonly occurs in various open habitats, while *I. alpestris* prefers forested areas (Van Buskirk, 2005). The second possible trade‐off is between competitive and dispersal ability. Under controlled laboratory conditions, *L. vulgaris* juveniles spontaneously walked longer distances than *I. alpestris* (Janča & Gvoždík, 2017). This suggests that the competitive disadvantage at the larval stage may be compensated for by a stronger dispersal after metamorphosis from other conspecific‐only metapopulations. Both hypothetical trade‐offs require empirical verification especially in applied ecology issues, for example, evaluating the possible impact of introduced *I. alpestris* on populations of native newt species (Arntzen, King, Denoel, Martinez‐Solano, & Wallis, 2016; Bell & Bell, 1995).

Although the competition‐predation trade‐off has been frequently reported as a mechanism allowing coexistence among ecologically similar species, our study provided no support for this scenario. We identified species body size as the potential factor changing the negative association to a positive one between these ecological performance traits. This concurs with the general importance of body size in aquatic communities (Hildrew, Raffaelli, & Edmonds‐Brown, 2007). Our study also highlighted two issues, which should be solved in further studies on competition‐predation trade‐off in newt or salamander larvae. First, it is virtually impossible to separate between the effect of exploitation and interference on larval growth rates. Second, it cannot be ruled out that our estimate of interference competition, injury rates, resulted from predation attempts rather than from aggression. This uncertainty may be problematic for some researchers but not for others, which consider intraguild predation as the mechanism of interference competition (Amarasekare, 2002; Wissinger et al., 1999). Despite these issues, interspecific interference competition and size‐selective predation are frequent species interactions, and so they deserve the same attention as exploitation competition in both theoretical and empirical studies on species’ coexistence within ecological communities.

## CONFLICT OF INTEREST

None declared.

## AUTHOR CONTRIBUTION

LG conceived and designed the experiments; MB, VK, and MH performed experiments; LG and MH analyzed data and wrote the manuscript. All authors approved and commented the final version.

## DATA ACCESSIBILITY

Competition and predation data are available at the Dryad Digital Repository: https://doi.org/10.5061/dryad.g59r413.
